# A 2‐year prospective clinical and bone density evaluation, with a subset undergoing radiostereometric analysis, using the Absolut cemented stem

**DOI:** 10.1111/ans.17519

**Published:** 2022-02-02

**Authors:** Jay R. Ebert, Nils O. Nivbrant, Victoria Petrov, Piers Yates, David J. Wood

**Affiliations:** ^1^ School of Human Sciences (Exercise and Sport Science) University of Western Australia Perth Western Australia Australia; ^2^ HFRC Perth Western Australia Australia; ^3^ Orthopaedic Research Foundation of Western Australia Perth Western Australia Australia; ^4^ Perth Orthopaedic Institute Perth Western Australia Australia; ^5^ Fremantle Hospital Fremantle Western Australia Australia; ^6^ School of Surgery (Orthopaedics) University of Western Australia Perth Western Australia Australia

**Keywords:** absolut, cemented, clinical outcomes, dual energy X‐ray absorptiometry, radiostereometric analysis, total hip arthroplasty

## Abstract

**Background:**

Total hip arthroplasty (THA) is common though the investigation of new prostheses requires a practical, step‐wise introduction. This study reports the 2‐year clinical results and periprosthetic bone mineral density (BMD) changes, along with a subset undergoing Radiostereometric analysis (RSA), in patients undergoing primary cemented THA using a new highly polished, double tapered, collarless femoral stem (Absolut).

**Methods:**

Between August 2013 and December 2016, 47 patients with a mean age of 74.2 years (range 36–89) underwent 51 THAs with the Absolut. All patients underwent clinical assessment pre‐surgery and at 6 weeks, 3, 12 and 24 months using the Oxford and Harris Hip Scores, as well as Dual Energy X‐ray Absorptiometry (DEXA) to assess BMD within 2–4 weeks post‐surgery, 12 and 24 months. RSA was undertaken in a patient subset (the first *n* = 30) early post‐surgery (1–2 days) and 3, 12 and 24 months.

**Results:**

All clinical scores significantly improved (*p* < 0.05). RSA revealed a mean subsidence of 0.78 mm at 3 months, 1.23 mm at 12 months and 1.51 mm at 24 months. Anterior–posterior and medial‐lateral translation was negligible. A significant increase (*p* = 0.020) in BMD was observed in Gruen zone 1, though no significant changes were observed for any other zone up until 2 years. Two patients acquired an early post‐operative deep vein thrombosis that were treated accordingly and resolved, with no further complications or re‐operations.

**Conclusion:**

The Absolut cemented femoral stem demonstrated good outcomes, BMD changes consistent with sound prosthesis integration and patterns of post‐operative micromotion observed in other successful cemented stems.

## Graphical abstract

Total hip arthroplasty (THA) is common though the investigation of new prostheses requires a practical, step‐wise introduction. In the current study, the Absolut cemented prosthesis, a highly polished, double tapered, collarless femoral stem (Absolut), demonstrated good outcomes, periprosthetic bone mineral density changes consistent with sound prosthesis integration and patterns of post‐operative micromotion observed in other successful cemented stems.

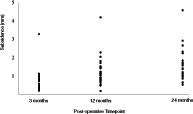

## Introduction

Total hip arthroplasty (THA) is one of the most common and effective operations for the treatment of arthritic hip disease with high levels of patient satisfaction.^1^ Polished, tapered, cemented femoral implants stems have demonstrated success in forming a stable mechanical interaction between the cement interface and bone, while permitting limited stem subsidence within the cement mantle, with this subsidence creating compressive forces in the adjacent cement that are transferred to bone as hoop stresses according to the ‘taper slip’ principle.^2^ The Exeter stem (Stryker, Mahwah, NJ), a collarless polished double tapered femoral prosthesis made of stainless steel, remains one of the most commonly used cemented femoral components with impressive and well‐reported survival rates,^3^, ^4^, ^5^, ^6^ as well as predictable subsidence patterns over time at the stem‐cement interface.^7^ Stefánsdóttir *et al*.^8^ reported a median distal migration of the Exeter of 1.34 mm and 1.77 mm at 2 and 5 years post‐surgery, respectively, evaluated via radiostereometric analysis (RSA). RSA is a highly accurate radiographic technique for assessing the stability and migration of the of the prosthetic implant,^9^, ^10^, ^11^ and it has been shown that stem migration in satisfactory implants is rapid initially and then slows,^12^ with those destined to fail early demonstrating rapid migration following this initial period.^13^

The Absolut stem (Global Orthopaedic Technology Pty Ltd., Sydney, Australia) is a highly polished, double tapered collarless cemented prosthesis with design parameters similar to that of the Exeter Stem. It was designed after analysis of the clinical and radiological data from 10 and 15 year series of Exeter and CPT (collarless polished tapered, Zimmer, Warsaw, Indiana) stems. In comparison to the successful Exeter, the Absolut stem has a smaller shoulder and is made of a cobalt chromium (CoCr) alloy rather than stainless steel, allowing it to accommodate larger offsets and theorized to enable the surgeon to better reproduce joint geometry and biomechanics. It had also been designed to optimize cement mantles and minimize the risk of mantle defects, which may be associated with failure.^14^, ^15^, ^16^ Unlike other stems, the Absolut stem has a true offset with no anthropomorphic variation, whereby the offset remains consistent and does not vary with increasing stem size. Hence, downsizing stems does not change offset, while allowing thicker and more complete cement mantles to be achieved easily. Compared with an offset range for the Exeter (range 31–50) and CPT (range 32–50) stems, the Absolut includes offsets of 37, 45 and 50, though without variation with stem size.

The aim of the current study was to evaluate the clinical outcome and periprosthetic bone change up until 2 years in a prospective series of patients undergoing primary THA for osteoarthritis with the Absolut cemented stem, together with an investigation of stem migration in a subset of the cohort. It was hypothesized that: (1) a significant improvement in clinical outcomes would be observed, (2) stem migration (and in particular stem subsidence) over the first 2 years would be similar to that reported for other successful cemented femoral stems and (3) no significant changes would be observed in periprosthetic bone over the evaluation period.

## Methods

**Fig. 1:**
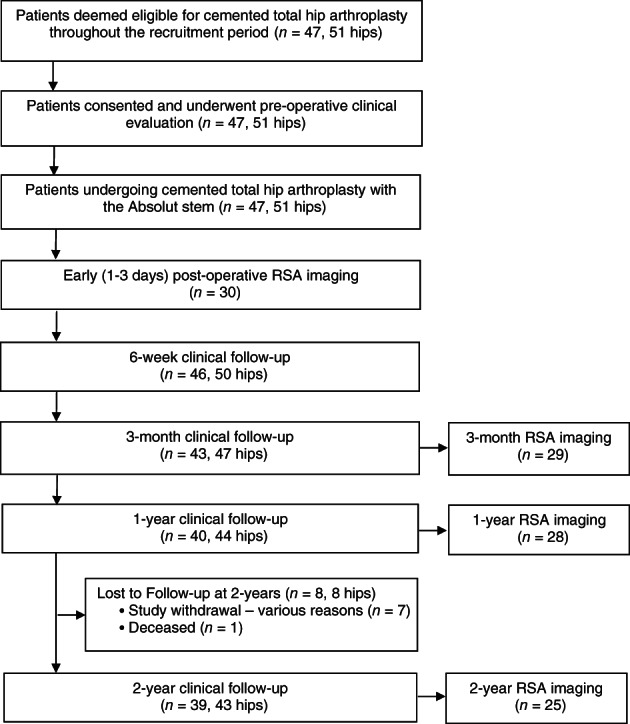
Flowchart demonstrating patient recruitment and follow‐up throughout the 2‐year post‐operative timeline.

Between August 2013 and December 2016, 47 patients (51 hips) underwent primary total hip arthroplasty (THA) with the Absolut cemented femoral stem, undertaken by a single high‐volume arthroplasty surgeon (DW) at a single private hospital. For the prospective clinical study follow‐up, exclusion criteria included age ≥90 years, a body mass index (BMI) >40, inflammatory arthritis, an active local or systemic infection, mental compromise (i.e., currently being treated for a psychiatric disorder, senile dementia, Alzheimer's disease, presence of alcohol and/or substance abuse), or an inability to provide informed consent and/or living outside of the metropolitan area. No patient that was a candidate for (and underwent) primary THA with the Absolut stem was excluded from study participation. Patient demographics are shown in Table 1, while Figure 1 demonstrates the flow of patient recruitment and follow‐up over the 2‐year post‐operative assessment timeline. Ethics approval was provided by the relevant hospital human research ethics committee (HREC), with the study undertaken according to the Declaration of Helsinki and the consent of all participants obtained prior to pre‐operative clinical review and subsequent THA surgery.

**Table 1:** Demographics of the patient sample that underwent surgery (*n* = 47, 51 hips)

Variable	Mean (Range)
Age (years)	74.2 (36–89)
Males (*n*,%)	15, 29.4%
Height (m)	1.66 (1.48–1.86)
Weight (kg)	71.4 (46.5–109.6)
Body Mass Index	25.9 (18.9–36.5)

### Operative technique and rehabilitation

All surgical procedures were performed using the minimally invasive, posterior approach with ‘tension dependent’ trans‐osseous repair of the short external rotator tendons. All patients underwent THA with the Absolut femoral stem (Global Orthopaedic Technology Pty Ltd., Sydney, Australia) and 32 mm diameter ceramic head and a cross‐linked polyethylene liner in either Trilogy or Continuum acetabular components (Zimmer, Warsaw, Indiana). Following surgery, all patients participated in a standardized post‐operative out‐patient rehabilitation program for a minimum of 6 weeks with a focus on early restoration of hip movement (within standard restrictions) and gait, followed by progressive strength and functional exercises with a focus on the restoration of functional capacity.

### Clinical outcome evaluation

Clinical assessment was undertaken pre‐surgery and at 6 weeks post‐surgery, as well as 3, 12 and 24 months. Two patient‐reported outcome measures (PROMs) were employed including the Harris Hip Score (HHS)^17^ and the Oxford Hip Score (OHS).^18^ The HHS is scored out of 100 (a higher score being desirable), including subscales of pain (0–44), function (0–47), deformity (0–4) and motions (0–5). The OHS is scored from 0 to 48 (a higher score being desirable), with this PROM primarily evaluating perceived hip pain and function. Objectively, patients had their maximal hip range of motion (ROM) assessed pre‐ and post‐surgery (on the operated limb only) using either a hand‐held bubble inclinometer (hip flexion in supine, internal and external rotation in prone) or a long arm goniometer (hip adduction and abduction in supine, extension in standing). The same qualified therapist with more than 20 years of clinical experience undertook all ROM measures, though no intra‐observer reliability assessment was undertaken.

### Dual energy X‐ray absorptiometry (DEXA) evaluation

DEXA was employed within the first 2–4 weeks post‐surgery (baseline) as well as 12 and 24 months post‐surgery, to evaluate bone changes during incorporation of the prosthesis using a Lunar Prodigy Vision DEXA machine (GE Medical Systems, Madison, WI). The periprosthetic bone mineral density (BMD, g/cm^2^) in the coronal plane was evaluated according to the seven Gruen zones.^19^

### Radiographic and radiostereometric analysis (RSA)

All patients underwent anteroposterior (AP) radiographs and the adequacy of cementing around the stem at 2‐years post‐surgery was determined as per the system reported by Barrack *et al*.^20^ The minimum mantle thickness was measured within each of the seven Gruen zones surrounding the stem. Bone resorption from the proximal medial femur was measured according to Engh *et al*.^21^

A subset of the cohort (the initial 30 patients) underwent RSA imaging within 1–3 days after surgery (30 hips) and at 3 (29 hips), 12 (28 hips) and 24 months (25 hips), using a standard protocol with an RSA calibration cage. At the time of surgery, 1 mm tantalum markers were inserted into the femur and cement, permitting evaluation of stem movement relative to the cement mantle. Translation of the femoral implant head centroid relative to the femur was calculated and reported in mm at 3, 12 and 24 months, in relation to the initial post‐operative (1–3 days) RSA films, using UmRSA 6.0 software (RSA Biomedical, Umeå, Sweden). Motion in all 3 planes was assessed: X medio‐lateral (medial positive), Y proximal‐distal (proximal positive) and Z antero‐posterior (anterior positive).

### Data and statistical analysis

Means (SD) were calculated for all measures throughout the pre‐ and post‐operative timeline, including clinical scores, DEXA values (as per the seven Gruen zones) and RSA movement (as per the three evaluated planes of motion). Medians (and range) were also calculated and presented for RSA measures. Repeated measures Analysis of Variance (ANOVA) was employed to evaluate the change in clinical scores, BMD and RSA‐assessed translation over time. *T*‐tests were employed to evaluate the magnitude of stem subsidence based on post‐operative Barrack grade (Barrack grade A versus other), while Spearman's correlation coefficient was employed to evaluate the association between stem subsidence and mean cement mantle thickness across the seven Gruen zones. Statistical analysis was performed using SPSS software (SPSS, Version 23.0, SPSS Inc., USA), while statistical significance was determined at *p* < 0.05. The sample size was initially determined based on prior studies employing RSA to evaluate stem movement. RSA studies have generally reported outcomes in small cohorts of 22–26 patients.^7^, ^8^, ^22^ Therefore, we sought to undertake an in‐depth RSA evaluation in the first 30 patients enrolled, with further recruitment (that excluded RSA evaluation) to *n* = 51.

## Results

As per Figure 1, 47 patients (51 hips) were recruited and underwent primary cemented THA with the Absolut prosthesis. Of these, post‐operative clinical review was undertaken in 46 patients (50 hips) at 6 weeks, 43 patients (47 hips) at 3 months, 40 patients (44 hips) at 12 months and 39 patients (43 hips) at 24 months. Specifically for the initial patient subset that underwent early post‐operative RSA investigation (30 patients, 30 hips), 29, 28 and 25 patients underwent subsequent RSA imaging at 3, 12 and 24 months, respectively (Fig. 1).

### Clinical results

A significant improvement (*p* < 0.05) was observed for all clinical scores (PROMs and hip range of motion measures) over the pre‐ and post‐operative timeline (Table 2).

**Table 2:** Clinical outcomes over the pre‐ and post‐operative timeline including the Oxford Hip Score (OHS), Harris Hip Score (HHS) and hip range of motion measures (degrees). Shown are means (SD)

Time‐point	OHS (0–48)	HHS (0–100)	Flexion	Extension	Abduction	Adduction	External rotation	Internal rotation
Pre‐surgery	24.9 (7.0)	54.3 (16.1)	93.0 (23.1)	13.4 (3.3)	26.1 (8.7)	11.6 (6.3)	23.0 (8.6)	20.4 (8.3)
6 weeks	36.9 (5.7)	80.6 (9.1)	92.5 (14.4)	15.5 (3.5)	30.2 (8.9)	14.0 (5.1)	27.4 (8.7)	27.9 (11.9)
3 months	43.5 (3.7)	91.9 (6.8)	108.0 (11.0)	17.6 (2.9)	33.9 (7.2)	17.7 (4.3)	27.7 (9.1)	34.2 (11.3)
12 months	45.9 (2.3)	94.0 (6.6)	112.0 (12.5)	18.0 (3.5)	38.5 (7.8)	19.8 (3.9)	30.4 (7.8)	38.0 (11.7)
24 months	46.2 (2.2)	95.2 (5.2)	112.4 (12.8)	18.9 (2.6)	39.0 (5.5)	21.1 (3.8)	29.7 (8.5)	35.5 (10.5)
p value	<0.0001	<0.0001	<0.0001	<0.0001	<0.0001	<0.0001	<0.0001	<0.0001

### Radiographic and RSA results

A total of 39 hips (76.5%) demonstrated complete cement mantles (Barrack grade A), with 11 hips (21.5%) demonstrating any full thickness mantle defect (Barrack grade C). One hip demonstrated a mantle that did not cover the tip of the stem (Barrack grade D). At 2‐year radiographic review, 50 hips (98%) had either no or first degree resorption of the proximal medial femoral cortex, with one hip demonstrating second degree.^21^ No significant difference (*p* = 0.510) was observed in 2‐year stem subsidence between those with Barrack grade A (1.58 mm) and C/D (1.45 mm), while no association was observed between stem subsidence and mean cement mantle thickness (*r* = 0.06, *p* = 0.341).

**Fig. 2:**
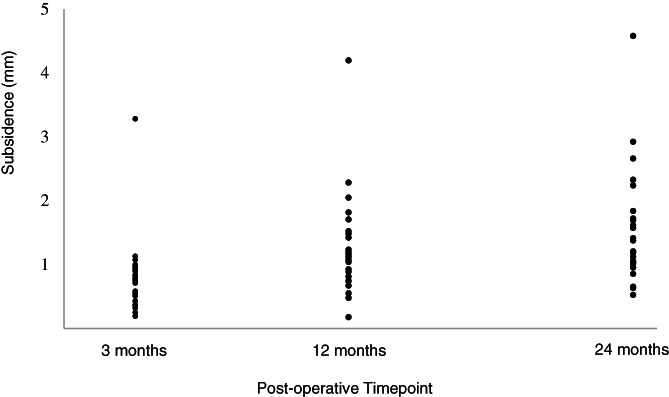
Stem subsidence (mm) evaluated via RSA for each of the Absolut stems at 3 (*n* = 29), 12 (*n* = 28) and 24 (*n* = 25) months post‐surgery.

RSA analysis of the Absolut stem revealed a mean subsidence of 0.78 mm at 3 months, 1.23 mm at 12 months and 1.51 mm at 24 months (Table 3), which was significant over time (*p* = 0.003). When excluding a single outlier which demonstrated subsidence of 3.28, 4.20 and 4.58 mm at 3, 12 and 24 months (Fig. 2), a mean subsidence across the full cohort of 0.69 mm, 1.12 mm and 1.38 mm was observed across the three time‐points. A non‐significant change (*p* = 0.956) in mean posterior translation of the prosthesis head of was observed with 0.58, 0.51 and 0.66 mm at 3, 12 and 24 months, respectively, while medial‐lateral translation was negligible (*p* = 0.559) (Table 3).

**Table 3:** Radiostereometric analysis (RSA) scores over the post‐operative timeline, including the change from 1 to 2 days to 3, 12 and 24 months post‐surgery. Shown are means (SD), medians and range

Post‐operative time‐point	Variable	Medial‐lateral (mm)	Proximal‐distal (mm)	Posterior–anterior (mm)
		Medial (+ve), Lateral (−ve)	Proximal (+ve), Distal (−ve)	Anterior (+ve), Posterior (−ve)
3 months (*n* = 29)	Mean (SD)	−0.07 (0.29)	−0.78 (0.55)	−0.58 (1.06)
	Median	−0.07	−0.74	−0.47
	Range	−0.69 to 0.83	−3.28 to −0.19	−3.71 to 2.50
12 months (*n* = 28)	Mean (SD)	−0.03 (0.45)	−1.23 (0.75)	−0.51 (1.45)
	Median	−0.06	−1.09	−0.37
	Range	−1.11 to 0.76	−4.20 to ‐0.18	−4.01 to 2.89
24 months (*n* = 25)	Mean (SD)	−0.19 (0.85)	−1.51 (0.96)	−0.66 (2.58)
	Median	−0.16	−1.38	−0.79
	Range	−2.67 to 2.19	−4.58 to −0.63	−7.63 to 8.28
*p*‐value		0.559	0.003	0.956

### DEXA results

With respect to the DEXA evaluation, a significant increase (*p* = 0.020) in BMD was observed in Gruen zone 1 (Table 4), though no significant changes (increase or decrease) were observed for any other zone up until 2 years, in comparison to the early DEXA assessment undertaken at 2–4 weeks post‐surgery.

**Table 4:** Bone mineral density (BMD, g/cm^2^) scores for each of the seven Gruen zones over the post‐operative timeline. Shown are means (SD)

Post‐operative time‐point	G1	G2	G3	G4	G5	G6	G7
2–4 weeks	1.23 (0.25)	2.19 (0.29)	2.35 (0.29)	2.47 (0.30)	2.33 (0.24)	1.97 (0.30)	1.36 (0.37)
12 months	1.29 (0.19)	2.17 (0.22)	2.33 (0.23)	2.43 (0.30)	2.31 (0.22)	1.98 (0.27)	1.35 (0.29)
24 months	1.44 (0.19)	2.33 (0.27)	2.52 (0.29)	2.68 (0.32)	2.48 (0.32)	2.11 (0.36)	1.33 (0.37)
*p*‐value	0.020	0.417	0.495	0.424	0.433	0.227	0.633

### Complications, adverse events, re‐operations and loss to follow‐up

Two patients in the cohort acquired a deep vein thrombosis (DVT), diagnosed at 3 and 6 weeks post‐surgery, respectively, both of which were treated accordingly and resolved without further sequalae. No further complications were observed. At 24 months post‐surgery, 17% of patients (*n* = 8) were lost to follow‐up (one deceased due to an unrelated illness, two patients were undergoing cancer treatment for a recent diagnosis, and five patients requested study termination due to time and travel constraints).

## Discussion

This is the first study that has reported clinical outcomes in patients undergoing primary THA with the Absolut stem, a highly polished, double tapered collarless cemented prosthesis. Good clinical outcomes over 2‐years were observed, along with a low complication and/or re‐operation rate, no evidence of significant periprosthetic bone change and subsidence and migration patterns similar to other reported successful cemented stems.

While the Absolut stem has design parameters similar to that of successful predecessors such as the Exeter^3^, ^4^, ^5^, ^6^ and the CPT stem,^23^, ^24^ it differs to the Exeter given its smaller shoulder and CoCr material. Compared with the Exeter stem which is made of stainless steel, the CoCr alloy accommodates a larger range of offsets, therefore better reproducing joint geometry and biomechanics. Yates *et al*. reported an 11% dislocation rate in their stainless steel CPT stem cohort (albeit in hips surviving beyond 10 years),^23^ though did suggest that once of the reasons may have been related to the limited range of offsets initially available for use. The Absolut stem had also been designed to optimize cement mantles and minimize the risk of mantle defects, which may be associated with stem failure.^14^, ^15^, ^16^ The current study demonstrated excellent mean clinical scores (HHS and OHS) at 1 and 2 years, with PROMs similar (or better) to those reported for other cemented stems such as the Exeter and CPT across various post‐operative time‐points.^24^, ^25^, ^26^, ^27^ A significant improvement over the pre‐ and post‐operative period was demonstrated, supporting the first hypothesis.

With cemented, polished, tapered stems it has been shown that stem migration in satisfactory implants is rapid initially and then slows.^12^ In the current study, a mean stem subsidence of 1.12 mm (median 1.09 mm) and 1.38 mm (median 1.38 mm) was observed in the first 1 and 2 years, respectively, after excluding a single outlier. Yates *et al*. reported subsidence of the CPT stem into the cement mantle of 0.44 mm/y, with 1.08 mm in the first year and 2.18 mm over 5 years, with subsidence associated with cement mantle defects.^25^ Stefánsdóttir *et al*. reported a median migration of the Exeter of 1.23 mm at 1 year, 1.34 mm at 2 years and 1.77 mm at 5 years,^8^ while Ling *et al*. reported long‐term subsidence of the Exeter of 1.83 mm (excluding outliers).^6^ Overall, the second hypothesis for the current study was largely supported with stem subsidence over the first 2 years similar to that reported for other successful cemented femoral stems, especially in considering previously reported subsidence for the Exeter and CPT stems.

There is generally good proximal bone preservation with tapered stems,^28^ with subsidence acting to distribute load more evenly throughout the entire stem‐cement and cement‐bone interface and reduce distal cement stresses.^25^, ^29^ DEXA has been reported as the reference standard method to evaluate BMD after THA.^30^, ^31^ In the current study, Gruen zone 1 demonstrated a significant increase in BMD over the 2‐year period, which could be explained by the greater offsets permitting more efficient hip abductor activity. However, no other significant zonal changes and certainly no reduction in BMD in proximal zones were observed. Damborg *et al*. reported a small but significant increase in BMD in Gruen zone 1 following THA employing the Exeter, though a significant reduction in zones 2, 3, 6 and 7, although no changes in zones 4 and 5.^32^ Broeke *et al*. reported a reduction in BMD over 2 years in all Gruen zones when comparing both the cemented SHP and Omnifit prostheses.^33^ In comparing the SHP and Lubinus SP2 cemented prostheses, Nivbrant *et al*. reported a reduction in BMD across all Gruen zones.^34^ In a study that sought to investigate whether femoral prosthetic geometry affected the pattern of strain‐adaptive bone remodelling in the proximal femur after THA, Jayasuriya *et al*. demonstrated that BMD loss was greatest in Gruen zone 7 (proximal medial femur) though similar in regional distribution and magnitude between the Charnley (composite‐beam), Exeter (double tapered) and C‐Stem (triple‐tapered) prostheses.^35^ Overall, the third hypothesis of the current study was partially supported, whereby apart form a small though significant increase in BMD in Gruen zone 1 over the 2‐year period, no other significant changes were observed in periprosthetic bone BMD.

A number of study limitations are acknowledged. First, this study included a small sample size and a cohort of patients that underwent surgery by a single experienced surgeon via a mini‐incision posterior approach. Therefore, while this approach (single surgeon) lacks generalizability across varied operators and surgical approaches, it may also be considered an important first step in investigating a new device to reduce variability that may present from these other sources. It was felt that the cohort was sound for a prospective pilot cohort of patients undergoing with a new prosthesis, and given the accuracy of RSA reported RSA studies generally report outcomes in small cohorts of 22–26 patients.^7^, ^8^, ^22^ Secondly, it should be acknowledged that patient and radiographic outcome should be continued well beyond the reported post‐operative 2 years, though the primary aims of the current study sought to report stem movement and periprosthetic bone changes over this initial period in a currently unreported prosthesis. Thirdly, we employed PROMs commonly reported in patients undergoing THA including the OHS and HHS,^17^, ^18^ though acknowledge that others exist and patient satisfaction was not specifically assessed.

## Conclusion

The Absolut cemented femoral stem demonstrated good clinical outcomes and no re‐operations within 2‐years, BMD changes consistent with sound prosthesis integration and patterns of post‐operative micromotion observed in other successful cemented stems. The Absolut stem (in comparison to successful predecessors such as the Exeter and CPT stems) differs due to its smaller shoulder and CoCr material, accommodating a larger range of offsets for the surgeon and the ability to better reproduce joint geometry and biomechanics. Ongoing patient and radiographic review are required to assess longer‐term migration patterns and failures beyond 2‐years.

## Conflict of interest

Global Orthopaedic Technology provided a research grant to assist this research project. There are no conflicts of interest associated with this publication and there has been no financial support for this work that could have influenced its outcome. Two authors (as outlined in the Cover Page) receive royalties for part‐development of the Absolut stem.

## Author contributions

**Jay R. Ebert:** Conceptualization; data curation; formal analysis; investigation; methodology; project administration; supervision; writing – original draft; writing – review and editing. **Nils O. Nivbrant:** Conceptualization; data curation; formal analysis; methodology; resources; software; writing – original draft; writing – review and editing. **Victoria Petrov:** Data curation; formal analysis; investigation; methodology; resources; writing – original draft; writing – review and editing. **piers yates:** Conceptualization; data curation; formal analysis; methodology; supervision; writing – review and editing. **David J. Wood:** Conceptualization; data curation; formal analysis; funding acquisition; investigation; methodology; project administration; supervision; writing – original draft; writing – review and editing.
